# Acute Renal Failure Induced by Chinese Herbal Medication in Nigeria

**DOI:** 10.1155/2015/150204

**Published:** 2015-06-25

**Authors:** Effiong Ekong Akpan, Udeme E. Ekrikpo

**Affiliations:** Department of Internal Medicine, University of Uyo Teaching Hospital, Uyo 520271, Nigeria

## Abstract

Traditional herbal medicine is a global phenomenon especially in the resource poor economy where only the very rich can access orthodox care. These herbal products are associated with complications such as acute renal failure and liver damage with a high incidence of mortalities and morbidities. Acute renal failure from the use of herbal remedies is said to account for about 30–35% of all cases of acute renal failure in Africa. Most of the herbal medications are not usually identified, but some common preparation often used in Nigeria includes “holy water” green water leaves, bark of *Mangifera indica* (mango), shoot of *Anacardium occidentale* (cashew), *Carica papaya* (paw-paw) leaves, lime water, *Solanum erianthum* (Potato tree), and *Azadirachta indica* (Neem) trees. We report a rare case of a young man who developed acute renal failure two days after ingestion of Chinese herb for “body cleansing” and general wellbeing. He had 4 sessions of haemodialysis and recovered kidney function fully after 18 days of admission.

## 1. Introduction

Several case reports in Europe, Asia, and China indicate increasing incidence of herbal medicine-induced nephrotoxicity [1, 2]. Traditional herbal medicine is a global phenomenon especially in the resource poor economy. Herbal medicines are used for treatments of different diseases, such as malaria, typhoid fever, infertility treatment, and for protection [3, 4] These herbal products are sometimes associated with complications such as acute kidney failure and liver damage with a high incidence of mortalities and morbidities [3]. Acute renal failure from the use of herbal remedies is said to account for about 30–35% of all cases of acute kidney failure in Africa [3, 5–7]. Most of the herbal medications are not usually identified, but some of the preparations often used in Nigeria include “holy water” green water leaves, bark of* Mangifera indica* (mango), shoot of* Anacardium occidentale* (cashew) leaves,* Carica papaya* (paw-paw) leaves, lime water,* Solanum erianthum,* (potato tree), and* Azadirachta indica* (Neem) tree [3, 8, 9].

## 2. Case Report

We report a case of a 30-year-old driver admitted through accident and emergency with history of haematuria and reduction in urinary output of 11- and 7-day duration, respectively.

Two days prior to the above complains, patient had ingested Chinese herbal tea for “general system cleansing” and generalized wellbeing following a few days of feeling unwell. The haematuria was painless and total. He took the tea for two days and the quantity each day was about 200 mLs. He had no bleeding from any other sites. He had no dysuria, no fever, no trauma to the abdomen, and no previous history of haematuria. He gave history of passage of less than 100 mLs of urine with associated facial and legs swelling. There was also history of vomiting and hiccups but no alteration in sensorium.

He was neither a known hypertensive nor diabetic patient and was not on any drug that is known to cause bleeding. He drank alcohol sparingly and was a nonsmoker.

On examination he was acutely ill, pale with facial swelling, and pitting leg oedema. He was not cyanotic and had no palpable peripheral lymph node enlargement. Pulse rate was 86 beats/minutes, with blood pressure of 130/70 mmHg. He had asterixis. Respiratory system and gastrointestinal tract were grossly intact.

His results of investigations were as follows:

HBsAg, anti-HCV, and HIV screening were all negative. Packed cells volume (PCV) = 23%. Urinalysis results were as follows: protein (+) and blood (+++). Sodium = 135 mmol/L (135–145), potassium = 4.3 mmol/L (3.2–5.0), bicarbonate = 21 mmol/L (22–28), phosphate = 0.85 (0.96–1.44) mmol/L, and calcium = 2.1 (2.1 = 2.6) mmol/L. Platelet = 230 × 10^9^/L. Urine microscopy was normal and culture yielded no growth after 24 hours of incubation. Fasting lipids profile was normal.

Liver function test was essentially normal, while renal ultrasonography revealed normal size kidney with preserved corticomedullary differentiation.

## 3. A Diagnosis of Acute Kidney Failure due to Chinese Herbal Medicine Was Made

He had 4 sessions of haemodialysis and erythropoietin injections. There was a progressive reduction in serum creatinine and urea from 1550 *μ*mmol/L and 49.32 mmol/L to 194 *μ*mmol/L and 10 mmol/L, respectively, on discharge (Table 1). Following these reductions and stability in urinary output, patient was discharged after 18 days of admission. He has remained stable 2 weeks after being discharged with a follow-up serum creatinine and urea result of 71 *μ*mmol/L and 3.4 mmol/L, respectively.

## 4. Discussion

Acute renal failure (ARF), now increasingly referred to as “acute kidney injury” (AKI), is characterized by a deterioration of renal function over a period of hours to days, resulting in the failure of the kidneys to excrete nitrogenous waste products and to maintain fluid and electrolyte homeostasis. This patient had haematuria 2 days after ingestion of Chinese herbal medication. Despite the widespread availability of dialysis, mortality rate for patients who developed ARF remains high, between 10%–50%, and this depends on the patient's comorbidities and the medical setting in which the kidney dysfunction occurs [10].

Studies in Nigeria have revealed that sepsis remained the leading cause of acute renal failure, accounting for as high as 38.8% in some studies [3]. Nephrotoxins from herbal medicine is another very important cause of acute renal in our environment [3, 8, 11]. Most of the herbal medications are not usually identified but some common ones include “holy water and green water [3, 4, 8, 9]. Leaves and bark of mango* (Mangifera indica) (mango),* shoot of* Anacardium occidentale* (cashew),* Carica papaya* (paw-paw) leaves, lime water,* Solanum eranthum* (potato tree) leaves and bark, and* Azadirachta indica* (Neem) tree leaves were identified as the commonest herbs in Ibadan [9]. Our index patient took Chinese herb for “body cleansing and general wellbeing.” Acute kidney injury associated with Chinese herbal medicine is common among the Asian population [12]. These herbal medicines often consist of complex substances with their synergistic effects resulting in kidney damage. The peculiarity of the kidneys such as its high vascularity, high cardiac output of 25%, and its large endothelial surface allowing for lots of toxins to be filtered makes it easily vulnerable to toxic injuries. The kidney injury may be diverse ranging from acute tubular necrosis, acute interstitial nephritis, fibrosis, chronic interstitial nephritis, malignancies, and several types of electrolytes disorders; the exact kidney lesion can only be made on biopsy. Unfortunately this was not done for the index patient because of nonacceptance.

His urine revealed haematuria but on microscopy there was no red blood cell. These findings suggest haemoglobinuria which may be a feature of mild to moderate intravascular haemolysis. The diagnosis of thrombotic microangiopathy was not considered because his platelet was normal; he had no fever and there was no purpura. The anaemia this patient had could have been because of the intravascular haemolysis or the haematuria although the amount of blood loss could not be quantified. Hypertension is not a common finding in patients with acute tubular disease except where there is fluid overload or increase rennin activity. This patient had only peripheral oedema; this may have been responsible for his normal blood pressure.

Mortality rates for patient requiring dialysis for acute renal failure range from 30% to as high as 80% [12, 13]. However 80% survival was recorded in patients in Ile Ife [11]. The evaluation and initial management of patients with acute kidney injury (AKI) should include the following: Assessment of the contributing causes of the kidney injury, an assessment of the clinical course including comorbidities, a careful assessment of volume status, and the institution of appropriate therapeutic measures designed to reverse or prevent worsening of functional or structural kidney abnormalities including withdrawal of the offending agent(s).

Optimization of the haemodynamic status and correction of any volume deficit will have a salutary effect on kidney function; it also helps minimize further extension of the kidney injury and potentially facilitate recovery. Our patient had a total of 4 sessions of intermittent haemodialysis with satisfactory recovery of renal function. This may be because he had no comorbidities. He was educated on the dangers posed by herbal medication before discharge.

## 5. Conclusion

In conclusion toxic renal injuries secondary to herbal medication are still common in our environment and with the high influx of Chinese herbal medicine, the trend is likely to continue. Public enlightenment is therefore necessary to educate the populace about dangers posed by herbal remedies.

## Figures and Tables

**Table 1:** Input-output chart and blood pressure.

Days on admission	Input (mls)	Output (mls)	Blood pressure (mmHg)	Creatinine (*µ*mmol/L)	Urea (mmol/L)
Day 1	1000	100	130/80	1352	49.32
Day 2	1000	100	130/80	1550	55.6
Day 3	1500	150	130/90		
Day 6	2000	1800	125/80		
Day 8	3500	3000	120/70	823	30.1
Day 10	5000	5300	130/70		
Day 11	5200	5600	130/75	497	27
Day 13	4300	6500	120/80		
Day 14	6500	5500	130/80	434	23.5
Day 15	4500	3900	120/85		
Day 17	4500	3500	120/80		
Day 18	4500	2500	120/80	194	10
